# 교대근무 경력 간호사의 식행동과 영향요인 분석: 2차자료 분석

**DOI:** 10.4069/kjwhn.2023.02.21.1

**Published:** 2023-03-02

**Authors:** Soyeon Kim, Jison Ki, Ji Yun Choi, Woan Heui Choi, Smi Choi-Kwon

**Affiliations:** 1 College of Nursing, Seoul National University, Seoul, Korea; 1 서울대학교 간호대학; 2 Research Institute of Nursing Science, Seoul National University, Seoul, Korea; 2 서울대학교 간호과학연구소; 3 Department of Nursing, Seoul National University Hospital, Seoul, Korea; 3 서울대학교병원 간호부분

**Keywords:** Diet, Feeding behavior, Nurses, Shift work schedule, 식이, 식행동, 간호사, 교대근무

## Abstract

**Purpose:**

This study investigated the dietary behavior of experienced shiftwork nurses and aimed to identify factors related to dietary behavior.

**Methods:**

This study was a secondary analysis based on the *Shift Work Nurses’ Health and Turnover* study (2018-2021) among Korean nurses. In total, 247 experienced (>12 months) shiftwork nurses were included in this study. The participants’ dietary behavior, depression, level of occupational stress, fatigue, physical activity, and general characteristics were measured. Descriptive statistics, Pearson correlation coefficients, independent t-test, one-way analysis of variance, the Kruskal-Wallis test, and multiple regression analysis were conducted.

**Results:**

The dietary behavior score of the participants using the Mini-Dietary Assessment Index was 29.35±5.67. Thirty percent of the participants were depressed, the participants experienced moderate occupational stress, and 74.1% of the participants engaged in an inadequate amount of physical activity. The factors influencing shiftwork nurses’ dietary behavior were having child(ren) (β=.16, *p*=.027), depression (β=–.13, *p*=.032), level of occupational stress related to occupational climate (β=–.13, *p*=.035), and an inadequate amount of physical activity (β=–.17, *p*=.006). These factors explained 10.4% of the variance in experienced shiftwork nurses’ dietary behavior scores.

**Conclusion:**

Experienced nurses with child(ren) tended to have healthier diets. However, a higher level of occupational stress related to occupational climate, depression, and engaging in an inadequate amount of physical activity were associated with a higher risk of having an unhealthy diet. Therefore, strategies are needed to encourage physical activity and alleviate adverse occupational climate and depression among experienced nurses.

## Introduction

개인의 건강을 유지하는 데에 적절한 영양 섭취는 매우 중요하다. 여성들은 남성들에 비해 건강한 식행동에 더 관심이 많고, 부적절한 식행동 및 그로 인해 발생하는 건강 문제들에 대하여 더 우려하는 경향이 있다[1]. 간호사는 대표적인 여성 직업 중 하나이나[2] 교대근무 간호사들은 식행동이 건강하지 않은 것으로 보고되었다[3]. 이는 과도한 업무 부담과 불규칙한 교대근무 스케줄이 원인이 될 수 있다[4]. 선행 연구 결과, 간호사들은 식사 시간이 불규칙하고, 결식 빈도가 높으며, 간식을 많이 섭취하고 식품 섭취가 불균형하기 쉬운 것으로 나타났다[3].

건강한 식행동이 고혈압[5], 당뇨[6]를 포함한 다양한 만성질환뿐만 아니라 암의 예방과도 관련이 있음은 여러 연구를 통하여 보고된 바 있다[7]. 우울 및 스트레스와 같은 부정적인 정서 상태[8]와 피로[9]는 건강한 식행동으로 인해 개선될 수도, 부적절한 식행동으로 인해 악화될 수도 있는 것으로 나타났다. 부적절한 식행동으로 발생할 수 있는 건강 문제들은 간호사의 이직 의도로도 이어질 수 있다[10]. 경력 간호사들의 이직 또한 간호의 질을 저하시키는 주요 요인으로 알려져 있어[11], 간호의 질에 있어서 경력 간호사의 중요성[12]을 고려하였을 때 경력 간호사의 건강 증진에 대한 관심이 필요하다. 따라서 경력 간호사의 건강 및 간호의 질을 증진하기 위한 한 가지 방안으로서 건강한 식행동은 중요하다고 할 수 있으며, 이에 경력 간호사의 건강한 식행동을 증진시키기 위한 연구가 필요하다.

선행 연구에서 교대근무 신규 간호사들의 식행동은 근무 기간, 우울, 피로, 스트레스에 영향을 받는 것으로 나타났다[3]. 경력 간호사들은 신규 간호사들에 비해 피로한 경향이 있으며[13], 더 심한 직무 스트레스를 경험하는 것으로 나타났다[14]. 직무 스트레스는 우울과 정적 상관관계를 갖는 것으로 보고되어 직무 스트레스가 심한 경력 간호사들은 더 우울할 가능성이 있는 것으로 보고되었다[15]. 선행 연구 결과, 직무 스트레스는 부적절한 식행동으로 이어질 수 있으며[16], 우울과 피로는 건강한 식행동을 저해하는 요소 중 하나인 것으로 나타나[3] 경력 간호사의 이러한 특성은 부적절한 식행동으로 이어질 위험이 있다. 간호사들의 대다수는 적정 수준의 신체활동을 하지 않는 것으로 나타났다[17]. 또한, 간호사들은 근무경력 1년이 지난 이후에 대학원을 진학하고자 하는 경우가 많아[18] 경력 간호사들은 신규 간호사에 비해 업무와 학업을 병행하거나, 가정 생활[19]을 병행하는 경우가 많으므로 이로 인한 시간 부족에 의해 신체활동이 감소할 수 있다. 신체활동이 적은 사람은 가공식품을 더 많이 섭취하고 채소 및 과일 섭취가 적은 것으로 보고되었으며[20], 꾸준한 운동은 보다 건강한 식품을 선호하도록 하는 것으로 나타났다[21]. 따라서 경력 간호사의 신체활동 감소 또한 부적절한 식행동으로 이어질 수 있다.

간호사의 식행동을 주요 개념으로 하는 선행 연구는 대부분 교대근무 간호사와 비교대근무 간호사를 비교하는 연구가 대부분으로[22], 교대근무 간호사 내에서 대상자의 특성에 따른 식행동의 차이를 조사한 연구는 찾아보기 힘들다. 또한, 선행 연구에서 신규 간호사의 식행동 및 그 영향요인은 종단연구로 조사된 바 있으나[3], 경력 간호사의 식행동과 이에 영향을 미칠 수 있는 요인에 대한 연구는 찾아보기 힘들었다. 이에 본 연구는 교대근무 경력 간호사의 식행동을 조사하고 근무경력 등 식행동에 영향을 줄 수 있는 요인과의 관계를 탐색하고자 한다.

본 연구의 목적은 다음과 같다.

첫째, 교대근무 경력 간호사의 식행동을 조사한다.

둘째, 교대근무 경력 간호사의 건강 문제 및 신체활동에 따른 식행동을 비교한다.

셋째, 교대근무 경력 간호사의 일반적 특성에 따른 식행동을 비교한다.

넷째, 교대근무 경력 간호사의 식행동에 영향을 미치는 요인을 파악한다.

## Methods

Ethics statement: Obtaining informed consent was exempted by the Institutional Review Board of Seoul National University (No. E2207/003-005) because this study was a secondary analysis using anonymized data.

### 연구 설계

본 연구는 2차자료 분석 연구로, 한국연구재단의 지원을 받아 2018-2021년에 시행된 Shift Work Nurses’ Health and Turnover (SWNHT) 코호트 연구를 원 자료로 한다[10]. 본 연구는 교대근무 간호사의 식행동을 조사하고, 그 관련 요인을 탐색하고자 시행한 횡단적 조사연구로, STROBE 보고지침에 따라 기술하였다(https://www.strobe-statement.org).

### 연구 대상

SWNHT 연구의 대상자는 서울 소재 2개 상급 종합병원의 신규 간호사와 경력 간호사로, 이때 신규 간호사(n=294)는 아직 근무 부서에 배치되지 않은 간호사들이었으며, 경력 간호사(n=300)는 조사 시점에서 근무 중인 간호사들이었다. SWNHT 연구에서는 월경 및 월경전증후군에 대한 조사가 포함되어 있었으므로 간호사 중 여성만을 대상으로 하였다. 신규 간호사는 총 세 차례(교대근무 전[T0], 교대근무 6개월 후[T1], 교대근무 18개월 후[T2])에 걸쳐 조사하였으며, 경력 간호사는 1차(T1) 조사 이후 12개월 뒤에 2차(T2) 조사를 진행하여 총 두 차례 조사하였다.

본 연구에서는 SWNHT 연구 자료 중 경력 간호사 T1 자료(n=300)를 이용하여 자료 분석을 시행하였다. 본 연구에서 경력 간호사는 임상 등급 중 초보자 단계를 넘은 2년차 이상의 간호사로 정의하였으며[23], 이에 따라 T1 자료 중 교대근무 기간이 12개월 이하인 대상자(n=49)와 주요 변수 응답에 누락이 있는 대상자(n=4)를 제외하였다. 이에 따라 본 연구의 총 대상자 수는 247명이었다.

본 연구에 필요한 최소 대상자의 수는 G*Power 3.1.9.7을 통하여 산출하였다. 다중 선형 회귀분석에 필요한 최소표본의 크기는 유의수준 .05, 검정력 .95, 중간 정도 효과크기 .15, 독립변수 5개를 기준으로 138명으로 도출하였다[24]. 본 연구의 대상자 수는 247명으로 연구에 필요한 최소 대상자 수를 만족시켰다.

### 연구 도구

#### 식행동

대상자의 식행동은 Kim 등[25]이 개발한 간이 식행동 진단표(Mini Dietary Assessment Index, MDA)를 이용하여 조사하였다. MDA는 5점 척도(‘전혀 아니다’ 1, ‘보통이다’ 3, ‘매우 그렇다’ 5)의 10개 문항으로 구성된 도구로, 일일 식사 횟수, 균형 식이 여부, 식사 시 간 추가 여부, 식품군에 따른 주관적 섭취 정도를 조사한다. 이때 조사되는 식품군에는 채소, 과일, 유제품, 아이스크림 및 가당 음료 등의 간식, 단백질, 고지방 식품, 튀긴 음식이 있다. 이때, 간식, 고지방 식품, 튀긴 음식의 섭취 정도는 역으로 환산하여 분석하였다. 점수 범위는 10–50점이며, MDA의 총점이 높을수록 식행동이 건강함을 의미한다. 그러나 MDA는 식품의 실제 섭취 빈도는 조사하지 않아 본 연구에서는 대상자의 각 식품군별 실제 평균 섭취 횟수를 추가 조사하였다. MDA 외에도 식사의 규칙성, 식사 속도, 주별 아침 식사 횟수 등을 추가 조사하였다.

#### 우울

본 연구는 대상자의 우울을 평가하기 위하여 Radloff [26]가 개발한 우울 척도(Center for Epidemiological Studies Depression Scale, CES-D)를 Kohout 등[27]이 20문항에서 10문항으로 축약한 축약형 CES-D의 국문 번안판[28]을 이용하였다. 축약형 CES-D는 4점 척도(‘잠깐 그런 생각이 들었거나, 그런 생각이 들지 않았음’ 0, ‘가끔 그런 생각이 들었음’ 1, ‘자주 그런 생각이 들었음’ 2, ‘항상 그런 생각이 들었음’ 3)로 그 점수 범위는 0–30점이며, 총점이 10점 이상일 때 우울 증상이 있는 것으로 해석한다[28]. 국내 연구에서 축약형 CES-D의 Cronbach’s α=.79였으며[28], 본 연구의 Cronbach’s α=.83이었다.

#### 직무 스트레스

대상자의 직무 스트레스는 Chang [29]이 개발한 한국형 직무 스트레스 측정도구 단축형(Korean Occupational Stress Scale, KOSS-26)을 이용하여 측정하였다. KOSS-26은 물리환경(2문항), 직무요구(4문항), 직무 자율성 결여(4문항), 관계갈등(3문항), 직무불안정(2문항), 조직체계(4문항), 보상부적절(3문항), 직장문화(4문항)의 8가지 영역, 26문항으로 구성된 도구로 4점 척도(‘전혀 그렇지 않다’ 1, ‘그렇지 않다’ 2, ‘그렇다’ 3, ‘매우 그렇다’ 4)로 평가한다. 직무 스트레스 총점은 8개의 하위 영역별 점수를 구한 다음(각 100점 만점) 이를 평균하여 산출하였다. 하위 영역별 점수는 개발자가 제시한 환산법을 이용하여 환산하였다. 총점의 범위는 0–100점으로, 점수가 높을수록 직무 스트레스가 높은 것을 의미한다[30]. KOSS-26의 개발 당시 Cronbach’s α=.82였으며[30], 본 연구의 Cronbach’s α=.80이었다. 도구 개발 당시 8가지 하위영역의 Cronbach’s α는 물리환경 .56, 직무요구 .71, 직무 자율성 결여 .66, 관계갈등 .67, 직무불안정 .61, 조직체계 .82, 보상부적절 .76, 그리고 직장문화가 .51이었다[29]. 본 연구에서의 8가지 하위영역의 Cronbach’s α는 물리환경 .42, 직무요구 .61, 직무 자율성 결여 .41, 관계갈등 .66, 직무불안정 .62, 조직체계 .69, 보상부적절 .60, 그리고 직장문화가 .61이었다.

#### 피로

본 연구에서는 대상자의 피로를 측정하기 위하여 Krupp 등[31]이 개발한 피로도 평가 척도(Fatigue Severity Scale, FSS)의 국문 번안판을 이용하였다[32]. FSS는 9문항, 7점 척도(‘전혀 그렇지 않다’ 1–‘매우 그렇다’ 7)로 이루어져 있다. 각 문항의 점수를 합산하여 평균을 낸 값의 범위는 1–7점으로, 평균이 4점 이상일 때 피로한 것으로 평가한다[31]. 도구 개발 시 원 도구의 Cronbach’s α=.89였으며[31], 본 연구의 Cronbach’s α=.93이었다.

#### 신체활동

본 연구에서 신체활동을 조사하기 위하여 사용한 도구는 World Health Organization (WHO)에서 개발한 국제신체활동설문지(Global Physical Activity Questionnaire, GPAQ) [33]의 국문 번안판[34]이다. GPAQ은 근무 중, 이동 시, 및 여가시간 신체활동량, 그리고 좌식생활 시간 등 4가지 영역을 평가하며, 총 16문항으로 구성되어 있다. 식행동과 신체활동의 관계를 조사한 기존 연구들에서 주로 일상생활에서의 신체활동량이 아닌 추가로 시행한 운동을 신체활동으로 분석함에 따라[21], 본 연구 또한 여가시간 신체활동량을 신체활동으로 분석하였다. 여가시간 신체활동량은 평소 여가시간에 시행하는 일주일 동안의 중강도 및 고강도 활동을 포함하며, 각각의 시행빈도와 시행시간을 토대로 ‘활동 형태에 따른 metabolic equivalent (MET) value×활동 시간(분)×주당 횟수’ 공식을 이용하여 METs-minute/week의 단위로 신체활동량을 계산하였다. MET value는 중강도 활동은 4.0, 고강도 활동은 8.0으로 계산하였다. 이때, 계산된 신체활동량의 값이 클수록 신체활동량이 많음을 의미한다[33]. WHO에서는 일반 성인에게 일주일에 75분 이상의 고강도 활동 또는 150분 이상의 중강도 활동 또는 이에 상응하는 수준의 신체활동을 하도록 권고하고 있다[35]. 이를 MET value를 이용하여 METs-minute/week의 단위로 신체활동량을 계산한 결과, 성인에게 권고되는 여가시간 신체활동량은 600 METs-minute/week였다. 이에 600 METs-minute/week 이상의 여가시간 신체활동을 하는 대상자는 적절한 수준의 신체활동을 하는 것으로 평가하였다.

#### 일반적 특성

본 연구에서는 대상자의 일반적 특성 파악을 위하여 연령(세), 키와 몸무게, 교육 정도(학사 이하/석사 이상), 결혼 여부(미혼/기혼), 자녀 유무, 근무 부서(일반 병동/중환자실/분만실), 근무경력(개월), 평균 밤근무 횟수(회/달)를 조사하였다. 조사한 키와 몸무게를 바탕으로 체질량지수(body mass index, BMI)를 계산하였으며, 대상자들의 근무경력을 토대로 상급 초보자 단계(입사 후 13–36개월), 유능 단계(입사 후 37–84개월), 그리고 숙련 단계(입사 후 85개월 이상)로 임상 등급을 분류하였다[23].

### 자료 수집

SWNHT 연구의 총 자료 수집 기간은 2018년 3월부터 2020년 4월까지이며, 경력 간호사에 대한 자료 수집은 1차 조사, 그리고 12개월 뒤에 시행된 2차 조사로 총 두 차례 시행하였다. 이때 1차 조사는 서면 설문지를 배부하였으며, 2차 조사 시에는 온라인 설문을 시행하였다. SWNHT 연구에서 조사된 변수는 일반적 특성, 이직 의도, 식행동, 월경 양상, 월경 전후기 증상, 혈액 및 체액 노출사고, 사회적 지지, 수면, 피로, 우울, 직무 스트레스, 신체활동, 프리젠티즘(presenteeism; 질병 등으로 인해 아픔에도 불구하고 출근을 하는 현상[36]) 등이었다. 본 연구에서는 그 중 일반적 특성, 식행동, 피로, 우울, 직무 스트레스와 신체활동 자료를 활용하였다. 본 연구를 위해 자료 활용에 대한 책임 연구자의 동의를 얻었으며, 본 연구에서는 SWNHT 연구 자료 중에서 두 차례의 경력 간호사 조사 중 1차 조사 자료를 이용하였다. 본 연구에 필요한 자료는 익명 처리된 상태로 전달받아 분석을 시행하였다.

### 자료 분석 방법

본 연구에서의 자료 분석은 IBM SPSS ver. 28.0 (IBM Corp., Armonk, NY, USA)을 이용하였으며, 통계적 유의 수준은 *p*<.05로 하였다. 사용된 도구의 신뢰도는 Cronbach’s α로 분석하였다. 대상자의 식행동, 우울, 직무 스트레스, 피로, 신체활동, 일반적 특성은 빈도, 백분율, 평균과 표준편차를 산출하였다. 식행동과 우울, 직무 스트레스, 피로, 신체활동, 그리고 일반적 특성 간의 관계를 알아보기 위하여 연속형 변수의 경우 Pearson 상관계수를 계산하였으며, 범주형 변수의 경우 독립 t검정, 일원분산분석, Kruskal-Wallis 검정을 이용하여 분석하였다. 또한, 식행동의 영향요인을 알아보기 위하여 입력 방법을 이용하여 다중회귀분석을 시행하였다.

## Results

### 식행동

대상자들의 평균 식행동 점수는 29.35±5.67점이었다(Table 1). 하루 평균 식사 횟수는 2.10±0.44회였으며, 균형 잡힌 식사 여부를 묻는 문항에서 49.8%가 ‘보통 균형 잡힌 식사를 한다’라고 대답하였다. 식사 시 소금이나 간장으로 간을 더 하는지 묻는 문항에서 167명(67.6%)이 ‘아닌 편이다’라고 응답하였다. 하루 평균 채소 섭취 횟수는 1.25±0.77회였으며, 과일 섭취 횟수는 0.78±0.61회였다. 유제품은 하루 평균 0.58±0.69회 섭취한 반면, 간식은 0.71±0.66회 섭취하는 것으로 나타났다. 또한, 콩과 고기 등 단백질 섭취 횟수는 하루 평균 1.55±0.78회였다. 고지방 식품을 섭취하는 횟수는 주당 평균 1.80±1.14회였으며, 튀긴 음식을 섭취하는 횟수는 주당 평균 2.11±1.29회였다.

MDA 외에 조사한 추가 문항을 분석한 결과, 식사가 불규칙적이라고 응답한 대상자는 234명(94.7%)이었으며, 식사에 소요되는 시간이 20분 미만으로 식사 속도가 빠른 대상자는 206명(83.4%)이었다. 대상자들의 평균 주당 아침식사 횟수는 1.93±1.86회였다.

### 건강 문제와 신체활동

대상자들의 건강 문제와 신체활동은 다음과 같다(Table 2). 우울 증상을 경험하는 대상자는 76명(30.8%)이었으며, 평균 직무 스트레스 점수는 50.56±9.12점으로 중등도 직무 스트레스를 느끼고 있었다. 또한 167명(67.6%)이 피로를 경험하는 것으로 나타났다. 대상자들의 평균 신체활동량은 504.78±1,001.23 METs-minute/week으로, 600 METs-minute/week 이상의 적절한 수준의 신체활동을 하는 대상자는 64명(25.9%)으로 나타났다.

### 일반적 특성

대상자의 일반적 특성은 다음과 같다(Table 3). 대상자들의 평균 연령은 29.30±4.59세였으며, 평균 BMI는 20.25±2.33 kg/m2였다. 학사 학위를 가진 대상자는 206명(83.4%)이었으며, 석사 과정에 재학 중이거나 그 이상의 학위 소지자는 41명(16.6%)이었다. 56명(22.7%)이 기혼이었으며, 자녀가 있는 대상자는 27명(10.9%)이었고, 198명(80.2%)이 일반 병동에서 근무하고 있었다.

대상자들의 평균 근무경력은 67.10±55.26개월이었으며, 상급 초보자 단계에 해당하는 대상자가 96명(38.9%)으로 가장 많았다. 대상자의 평균 밤근무 횟수(회/달)는 6.18±1.02회였다.

### 건강 문제 및 신체활동에 따른 식행동

우울한 대상자는 그렇지 않은 대상자보다 식행동이 부적절한 것으로 나타났으며(t=2.93, *p*=.004), 부적절한 수준의 신체활동을 하는 사람은 적절한 신체활동을 하는 사람에 비해 식행동이 부적절한 것으로 나타났다(t=2.95, *p*=.003). 그러나 식행동은 피로 여부에 따른 유의한 차이를 보이지 않았다(Table 4).

대상자들의 직무 스트레스 총점은 식행동과 유의한 상관관계를 보이지 않았으나 KOSS-26의 8개의 하위 영역 중 ‘직장문화 직무 스트레스’는 건강한 식행동과 약한 부적 상관관계를 보였다(r=–.19, *p*=.003) (Table 5).

### 일반적 특성에 따른 식행동

대상자의 식행동은 학력 및 결혼 여부에 따른 유의한 차이를 보이지 않았으나 자녀가 있는 경우 더 건강한 식행동을 하였다(t=–2.90, *p*=.004). 대상자의 식행동은 근무지에 따른 차이를 보이지 않았으나 대상자의 식행동은 임상 등급에 따라 유의한 차이를 보였다(F=3.62, *p*=.028) (Table 4). 추가로 사후분석을 한 결과, 유능 및 숙련 단계의 대상자는 상급 초보자 단계의 대상자에 비해 더 건강한 식행동을 하는 것으로 나타났다. 그러나 유능 단계와 숙련 단계의 대상자 사이의 식행동에는 유의한 차이가 없었다.

일반적 특성 중 연속 변수들에 관하여 식행동과의 상관관계를 조사한 결과, 간호사들의 건강한 식행동은 연령과 약한 정적 상관관계가 있는 것으로 나타났다(r=.19, *p*=.003). 그러나 BMI는 식행동과 유의한 상관관계를 보이지 않았다. 근무기간은 건강한 식행동과 약한 정적 상관관계를 보였으나(r=.19, *p*=.003), 밤근무 횟수는 식행동과 유의한 상관관계가 없는 것으로 나타났다(Table 5).

### 식행동의 영향요인

교대근무 간호사의 식행동에 영향을 미치는 요인을 파악하기 위하여 다중회귀분석을 시행하였다(Table 6). 이때 종속변수는 MDA 총점으로 설정하였으며, 식행동과 유의한 관계가 있었던 자녀 유무, 근무경력, 우울, 직장문화 직무 스트레스, 그리고 신체활동을 독립변수로 하였다. 이때 자녀 유무, 우울, 신체활동은 범주화된 변수로서 각각 ‘자녀 무’, ‘우울 무’, ‘적절 수준의 신체활동’을 기준으로 가변수 처리를 시행하였다. 통제변수로서의 대상자의 연령은 근무경력과의 피어슨 상관계수가 .97로 근무경력과 매우 높은 상관관계를 보여 분석에서 제외하였다. 분석 방법은 입력 방법을 활용하였다. 회귀모형의 적합도는 F=6.69, *p*<.001로 적합한 것으로 나타났으며, Durbin-Watson값 또한 2.03으로 잔차의 독립성 가정을 만족하는 것으로 나타났다. 회귀모형의 설명력은 10.4%였다. 다중회귀분석 결과, 대상자들의 건강한 식행동에 영향을 주는 요인들은 신체활동(β=–.17, *p*=.006), 자녀의 유무(β=.16, *p*=.027), 우울(β=–.13, *p*=.032), 직장문화 직무 스트레스(β=–.13, *p*=.035)였다.

## Discussion

본 연구는 서울 소재의 상급 종합병원 두 곳에서 근무하는 247명의 경력 간호사들을 대상으로 식행동 및 식행동의 영향요인을 조사하였다. 기존 연구들에서는 간호사들의 식행동에 영향을 주는 요인으로 교대근무를 주로 다루었으나[22], 본 연구에서는 교대근무를 포함하여 식행동에 영향을 미칠 수 있는 다른 요인들도 탐색하고자 하였다.

본 연구 대상자들의 평균 식행동 점수는 29.35점으로, 도구 개발 시에 건강한 성인의 식행동 점수로 추정된 30점보다 낮은 것으로 나타났으며[25], 국내 선행 연구에서 일반적인 여성들의 평균 식행동 점수였던 30.3점에 비해서도 더 낮았다[37]. 이는 교대근무 간호사들이 정작 자신의 건강증진 행위에는 소홀하며[38], 식행동이 건강하지 않다는 선행 연구를 지지한다[39]. 그러나 신규 간호사의 식행동과 비교하였을 때에는 경력 간호사인 본 연구 대상자의 식행동이 더 건강한 것으로 나타났다[3]. 본 연구에서 식행동 점수는 근무경력과 유의한 정적 상관관계를 보여 근무경력이 많아질수록 건강한 식행동을 하는 것으로 보인다. 근무경력과 식행동의 유의한 상관관계는 여러 가지로 해석이 가능하다. 첫째, 경력 간호사의 업무 능력이 향상되어 근무 중 식사를 할 여유가 생기고, 정시 퇴근이 가능하여 식사 시간이 확보되었을 수 있다[40]. 둘째, 경력 간호사의 경우, 신규 간호사보다 기혼자가 많고 자녀가 있는 대상자가 많아 이러한 요인이 식행동에 긍정적인 영향을 미쳤을 수 있다.

본 연구에서도 다중회귀분석 결과, 자녀의 유무는 식행동에 영향을 미치는 유의한 영향요인이었다. 추가 분석을 시행한 결과, 본 연구에서 자녀가 있는 대상자는 그렇지 않은 대상자보다 식사를 비교적 규칙적으로 하며, 채소 및 단백질 섭취 빈도가 유의하게 높았다. 이는 자녀가 있는 여성이 자신의 식행동이 자녀에게 미칠 영향을 고려하여 건강한 식행동에 더 관심을 가져 나타난 결과일 수 있다[41].

본 연구 결과, 부적절한 수준의 신체활동은 식행동의 영향요인이었다. 이러한 연구 결과는 규칙적인 운동을 하는 사람의 식사의 질이 그렇지 않은 사람의 식사의 질보다 우수하다는 기존 국외 연구 결과와 유사하다[42]. 추가분석 결과, 부적절한 수준의 신체활동을 하는 사람은 과일 및 단백질 섭취 횟수가 적절한 수준의 신체활동을 하는 사람에 비해 더 적은 것으로 나타났다. 이는 신체활동량이 적은 사람이 신체활동량이 많은 사람보다 과일 섭취량[43] 및 단백질 섭취량[44]이 적다는 기존 연구와 유사하다. 그러나 다른 선행 연구에서는 건강한 식행동을 하는 사람이 그렇지 않은 사람보다 신체활동량이 많다는 역방향의 결과가 나타났다[45]. 기존 연구 결과에 따르면 신체활동은 건강한 식행동을 포함한 건강증진 행위를 촉진하는 요소로 밝혀졌으며[46], 역으로 건강한 식행동은 신체활동을 포함한 건강증진 행위를 촉진하는 요소로 나타났다[47]. 본 연구에서는 간호사의 건강증진 행위에 대하여 따로 조사하지 않아 정확히 알 수는 없으나, 이러한 결과들은 건강한 식행동과 신체활동과 같은 건강증진 행위를 실천하는 사람은 건강에 대한 관심이 있으며, 이러한 관심이 다른 건강증진 행위의 수행으로 이어져 나타난 결과일 수 있다.

대상자들의 직장문화 직무 스트레스는 식행동의 유의한 영향요인으로 나타났다. 부정적인 직장문화는 비효율적인 업무 처리로 이어져 간호사들의 업무량 증가 및 휴게시간 부족으로 이어질 수 있으며[48], 간호사들의 근무 중 결식, 또는 속식과 같은 부적절한 식행동을 유발할 수 있다[49]. 본 연구에서 높은 직장문화 직무 스트레스는 간식 섭취 증가와 정적인 상관관계를 보였다. 아직까지 직장문화 직무 스트레스와 식행동의 관련성에 대한 연구가 부족하여 정확한 이유는 확인할 수 없으나 높은 직무 스트레스가 개인의 충동 조절 능력을 감소시키고[50], 당에 대한 요구도를 높여[51] 나타난 결과일 수 있다. 본 연구 결과에서 직장문화 직무 스트레스는 다른 직무 스트레스 하위 영역에 비해 상대적으로 낮은 편이어서 그 중요성이 적어 보이기도 하나, 간호사들의 식행동 영향요인으로서 중요할 수 있음을 시사한다.

본 연구 결과, 우울한 대상자는 부적절한 식행동을 할 위험이 더 큰 것으로 나타났다. 이는 적은 채소와 과일 섭취[52], 그리고 잦은 결식 및 간식 섭취의 증가[53]와 관련이 있을 수 있다. 본 연구 대상자들 중 우울 증상을 경험하는 대상자는 그렇지 않은 대상자들에 비하여 하루 평균 식사 횟수가 적고, 간식 섭취 횟수가 많은 것으로 나타났다. 그러나 추가 분석을 시행한 결과, 본 연구에서는 우울 여부에 따른 채소 및 과일 섭취 빈도의 차이를 보이지 않았다. 이는 본 연구 대상자들의 전체적인 채소 및 과일 섭취 빈도가 현저히 낮아 우울 여부에 따른 차이를 보이지 않아 나타난 결과일 수 있다. 2020년 발표된 한국인 영양소 섭취 기준을 바탕으로 발표된 여성의 일일 채소와 과일 섭취 횟수는 각각 8회, 2회이나[54] 본 연구 대상자들 중 채소를 권장 섭취 횟수만큼 섭취하는 대상자는 없었으며, 과일을 권장 섭취 횟수만큼 섭취하는 대상자는 22명(7.5%)에 불과하였다.

선행 연구들에서 피로와 우울은 밀접한 관련이 있는 것으로 나타났으며[55], 신규 간호사의 식행동을 조사한 기존 종단연구에서 피로는 우울과 함께 신규 간호사의 부정적인 식행동의 영향요인인 것으로 보고하였다[3]. 그러나 본 연구 결과에서는 피로 여부에 따른 식행동의 차이를 보이지 않았다. 이러한 결과의 차이는 정확하게 알 수는 없으나 경력 간호사가 신규 간호사보다 피로한 경향이 있어 피로가 식행동에 유의한 차이를 주지 못해 나타난 결과일 수 있다[13]. 또한, 추가 분석 결과, 피로하지 않은 군이 피로한 군보다 간식 섭취가 더 많은 것으로 나타났다. 이는 예상치 못한 결과로 기존 연구와 상반되는 결과였다[56]. 그러나 이는 아마도 간식 섭취를 통해 피로가 감소하여 나타난 결과일 수 있다[57]. 특히 하루 평균 식사 횟수가 2.1회에 그친 본 연구 대상자들을 고려하였을 때, 오히려 대상자들이 간식 섭취를 통해 부족한 에너지 섭취를 보충하였을 수 있고[58], 이것이 피로를 예방하는 효과를 주었을 수 있다.

본 연구의 제한점은 다음과 같다. 첫번째로 본 연구에서 간호사 식행동에 대한 회귀분석 결과의 설명력은 10.4%였다. 식행동 조사는 간호사뿐만 아니라 일반인을 대상으로 하여도 연구 수행이 어려워 선행 연구를 찾기 힘들었고, 특히 식행동의 영향요인을 조사한 연구는 찾기는 더 어려워 연구 결과의 설명력을 비교하는 데에 있어 한계가 있었다. 본 연구의 낮은 설명력은 식행동은 개인의 신체적, 정신적 건강 상태뿐만 아니라 주변 환경 및 경제 상태 등 다양한 요인들로부터 영향을 받을 수 있으나[59] 본 연구는 간호사의 건강을 조사한 SWNHT 연구의 2차분석 연구로 파악할 수 있었던 요인의 수가 제한적이어서 나타난 결과일 수 있다. 따라서 간호사의 식행동의 영향요인을 총체적으로 분석할 수 있는 연구를 추가로 수행할 필요가 있다. 또한, 본 연구에서 사용한 도구인 MDA는 각 문항이 각기 다른 식행동을 조사하므로 도구 개발 시부터 신뢰도가 제시되지 않았다는 제한점이 있으나, 자가 보고 형식으로 대상자가 응답이 용이하다는 점에서 모 연구에서 사용하였다. 이에 각 식품군별 식품의 섭취 빈도를 추가 조사하여 이러한 제한점을 보완하고자 하였다. 두 번째로 본 연구에서의 직무 스트레스의 각 영역별 신뢰도는 .41–.69으로 비교적 낮은 신뢰도를 보였다. 간호사를 대상으로 시행된 선행 연구에서도 직무 스트레스 하위 영역의 각 영역별 신뢰도가 .48–.78로 낮은 신뢰도를 보였다[60]. 이처럼 낮은 신뢰도는 간호사의 직업적 특성과 각 대상자의 부서별 특성 및 개인적 특성의 차이에 의해 발생한 것일 수 있다. 특히 신뢰도가 낮게 나타난 ‘직무 자율성 결여’ 영역의 경우, 선행 연구에서도 하위영역 중 가장 낮은 신뢰도를 보였다[60]. 이는 부서의 특성에 따라 업무를 수행하기 위한 기술 및 지식의 수준이 다를 수 있고, 개인의 경력 및 부서 내 위치에 따라 업무량 및 업무 수행 과정의 조절 여부 및 정도가 달라질 수 있기 때문에 내적 일관성이 더 떨어져 나타난 결과일 수 있다. 또한, KOSS-26은 도구 사용에 대한 전문 교육을 이수한 보건 관리자의 책임 하에 설문을 진행하도록 권고하나[30] SWNHT 연구에서는 자가 응답식으로 진행하였다는 점이 신뢰도에 영향을 주었을 수 있다. 이에 추후 연구에서는 간호사의 직업적 특성 및 개인적 특성을 고려한 도구의 개발과 도구의 수정 및 보완이 필요할 것이다. 마지막으로 본 연구의 대상자들은 모두 상급 종합병원에 1년 이상 근무한 간호사로, 다른 환경에서 근무하는 간호사에 대한 조사는 이루어지지 않아 전체 간호사 집단에 연구 결과를 적용하기는 어려울 수 있다. 그러나 본 연구는 식사 횟수, 식품군별 섭취 횟수 등 경력 간호사들의 식행동 전반을 조사하고, 신체적, 정신적 건강 상태를 토대로 영향요인을 규명하고자 하였다는 점에서 그 의의가 있다.

본 연구에서 교대근무 경력 간호사들의 식행동은 일반 성인에 비하여 다소 부적절하였다. 또한 약 30%가 우울을 경험하고, 중등도의 직무 스트레스가 있는 것으로 나타났다. 또한, 약 74%의 간호사의 신체활동량이 부적절하였다. 교대근무 경력 간호사들의 식행동은 자녀의 유무, 신체활동, 직장문화 직무 스트레스, 우울에 영향을 받는 것으로 나타났으며, 자녀가 있을 때 부적절한 식행동을 할 위험이 적었다. 그러나 직장문화 직무 스트레스가 높고, 우울하며, 부적절한 수준의 신체활동을 하는 경우 식행동이 부적절할 위험이 증가하였다. 따라서, 간호사들의 신체활동량을 증가시킬 수 있는 건강증진 프로그램을 제공하고, 직장문화를 개선하며, 우울을 완화시키기 위한 전략을 수립하는 등 조직적 노력이 필요함을 제언한다.

## Figures and Tables

**Table 1.** Dietary behavior of the participants (N=247)

MDA index	Categories	n (%) or mean±SD
MDA score^†^		29.35±5.67
Meals (times/day)		2.10±0.44
Balanced diet	Always	69 (27.9)
	Sometimes	123 (49.8)
	Rarely	55 (22.3)
Additional salt	Always	13 (5.3)
	Sometimes	67 (27.1)
	Rarely	167 (67.6)
Food intake	Vegetable (times/day)	1.25±0.77
	Fruit (times/day)	0.78±0.61
	Dairy (times/day)	0.58±0.69
	Snack (times/day)	0.71±0.66
	Protein (times/day)	1.55±0.78
	High fat (times/week)	1.80±1.14
	Fried food (times/week)	2.11±1.29

MDA: Mini-Dietary Assessment index.

† Possible range, 10–50.

**Table 2.** Health problems and physical activity of the participants (N=247)

Variables	Categories	Possible range	n (%)	Mean±SD
Depression	Total	0–30		7.93±4.79
	Nondepressed (<10)		171 (69.2)	5.35±2.40
	Depressed (≥10)		76 (30.8)	13.75±3.53
Occupational stress	Total	0–100		50.56±9.12
	Job demands	0–100		77.80±13.81
	Physical environment	0–100		69.70±16.90
	Lack of reward	0–100		54.97±16.25
	Organizational system	0–100		51.92±16.31
	Insufficient job control	0–100		50.24±12.76
	Occupational climate	0–100		35.86±16.15
	Interpersonal conflict	0–100		33.51±14.55
	Job insecurity	0–100		30.43±23.27
Fatigue	Mean	1–7		4.47±1.15
	Non-fatigue (<4)		80 (32.4)	3.16±0.60
	Fatigue (≥4)		167 (67.6)	5.10±0.76
Physical activity (METs-minute/week)	Total			504.78±1,001.23
	Adequate (≥600)		64 (25.9)	
	Inadequate (<600)		187 (74.1)	

MET, Metabolic equivalent.

**Table 3.** General characteristics of the participants (N=247)

Variable	Categories	n (%) or mean±SD
Age (year)		29.30±4.59
Body mass index (kg/m^2^)		20.25±2.33
Level of education	≤Bachelor	206 (83.4)
	≥Master	41 (16.6)
Marital status	Unmarried	191 (77.3)
	Married	56 (22.7)
Have child(ren)	No	220 (89.1)
	Yes	27 (10.9)
Workplace	General ward	198 (80.2)
	Intensive care unit	47 (19.0)
	Delivery room	2 (0.8)
Total working period (month)		67.10±55.26
Clinical ladder stage^†^	Advanced beginner	96 (38.9)
	Competent	84 (34.0)
	Proficient	67 (27.1)
Night shift (times/month)		6.18±1.02

† The clinical ladder stage was defined according to the total working period: advanced beginner (13–36 months), competent (37–84 months), and proficient (over 85 months).

**Table 4.** Dietary behavior by characteristics (N=247)

Variable	Categories	Mean±SD	t or F	*p*
Depression	Nondepressed (<10)	30.05±5.46	2.93	.004
	Depressed (≥10)	27.79±5.87		
Fatigue	Non-fatigue (<4)	30.30±5.60	1.83	.069
	Fatigue (≥4)	28.90±5.67		
Physical activity	Adequate (≥600)	31.13±5.67	2.95	.003
(METs-minute/week)	Inadequate (<600)	28.73±5.56		
Level of education	≤Bachelor	29.07±5.59	–1.77	.078
	≥Master	30.78±5.96		
Marital status	Unmarried	28.98±5.49	–1.89	.060
	Married	30.61±6.16		
Have child(ren)	No	28.99±5.42	–2.90	.004
	Yes	32.30±6.88		
Workplace	General ward	29.13±5.47	2.30^‡^	.316
	Intensive care unit	30.38±6.36		
	Delivery room	27.00±8.90		
Clinical ladder stage^†^	Advanced beginner	28.31±5.34	4.31^§^	.015
	Competent	29.29±5.29		
	Proficient	30.93±6.30		

MET, Metabolic equivalent.

† The clinical ladder stage was defined according to the total working period: advanced beginner (13–36 months), competent (37–84 months), and proficient (over 85 months).

‡ Kruskal-Wallis test;

§ one-way ANOVA.

**Table 5.** Relationships between dietary behavior and participants’ characteristics (N=247)

Variable	Categories	MDA score	
		r	*p*
Occupational stress	Total	–.11	.098
	Job demands	–.01	.872
	Physical environment	–.03	.700
	Lack of reward	–.09	.162
	Organizational system	–.06	.338
	Insufficient job control	–.10	.132
	Occupational climate	–.19	.003
	Interpersonal conflict	–.05	.454
	Job insecurity	–.00	.963
Age		.19	.003
Body mass index		.10	.122
Total working period (month)		.19	.003
Night shifts (times/month)		.07	.266

MDA, Mini-Dietary Assessment index.

**Table 6.** Factors influencing dietary behavior (N=247)

Variable	Categories	B	SE	β	t	*p*
(constant)		32.44	1.13		28.74	<.001
Depression^†^		–1.64	0.76	–.13	–2.15	.032
Occupational stress	Occupational climate	–0.05	0.02	–.13	–2.12	.035
Physical activity^†^		–2.21	0.80	–.17	–2.79	.006
Total working period		0.06	0.08	.06	0.80	.426
Having child(ren)^†^		2.97	1.34	.16	2.23	.027

B: Unstandardized coefficients; β: Standardized coefficients.

† Reference groups were depression (nondepressed), physical activity (adequate), and having child(ren) (no).
